# Prophylaxis and treatment of influenza: options, antiviral susceptibility, and existing recommendations

**DOI:** 10.3205/id000071

**Published:** 2021-04-30

**Authors:** Susanne C. Duwe, Barbara Schmidt, Barbara C. Gärtner, Jörg Timm, Ortwin Adams, Helmut Fickenscher, Michaela Schmidtke

**Affiliations:** 1 Robert Koch Institute, Unit 17: Influenza and Other Respiratory Viruses, National Reference Centre for Influenza, Berlin, Germany; 2 Institute for Clinical Microbiology and Hygiene, Regensburg University Hospital, Regensburg, Germany; 3 Institute of Medical Microbiology & Hygiene, Saarland University Medical Center, Homburg, Germany; 4 Institute for Virology, University Hospital Düsseldorf, Faculty of Medicine, University Düsseldorf, Germany; 5 Institute for Infection Medicine, University of Kiel and University Hospital Schleswig-Holstein, Kiel, Germany; 6 Section Experimental Virology, Department of Medical Microbiology, Jena University Hospital, Germany

**Keywords:** infection, neuraminidase, baloxavir, polymerase, resistance

## Abstract

Influenza viruses of types A and B attack 5–10% of adults and 20–30% of children, thereby causing millions of acute respiratory infections in Germany annually. A significant number of these infections are associated with complications such as pneumonia and bacterial superinfections that need hospitalization and might lead to death. In addition to vaccines, drugs were developed that might support influenza prevention and that can be used to treat influenza patients. The timely application of anti-influenza drugs can inhibit virus replication, help reduce and shorten the symptoms, and prevent death as well as virus transmission.

This review concisely describes the mechanism of action, the potential for prophylactic and therapeutic use, and the knowledge on resistance of anti-influenza drugs approved today. However, the main aim is to give an overview on the recommendations available in Germany for the proper use of these drugs. In doing so, the recommendations published in statements and guidelines of medical societies as well as the German influenza pandemic preparedness plan are summarized with the consideration of specific circumstances and groups of patients.

## 1 Introduction

Influenza is a serious and frequently underestimated viral disease. For example, 25,100 influenza-associated deaths and 9 million influenza-attributable acute respiratory illnesses of patients seeking medical attention were estimated for Germany in the 2017–2018 season [1]. Although the influenza season 2018–2019 was characterized by significantly lower case numbers, 3.8 million patients seeking medical attention for influenza-attributable acute respiratory illnesses, 2.3 million incapacities for work, and 18,000 influenza-related hospitalizations were estimated [2]. These data confirm the known high influenza attack rates (5–10% in adults and 20–30% in children) [1].

Most infections with influenza A and B viruses are asymptomatic or result in acute self-limiting uncomplicated influenza with sudden onset of fever (≥38.5°C), dry cough, sore throat, body aches and pains, and headaches. Fatigue, sweating, rhinorrhea, as well as nausea, vomiting, and diarrhea were reported as additional symptoms. In the absence of complications and risk factors, an influenza illness lasts 5–7 days [3]. Influenza complications that prolong the disease and increase the severity of symptoms include pneumonia, bacterial superinfection, and a postinfectious elevated risk of myocardial infarctions as well as stroke associated with the influenza disease. Such complications need hospitalization of influenza patients and might cause more than 20,000 deaths as seen for example in Germany in the 2014–2015, 2016–2017, and 2017–2018 influenza seasons [1].

Although non-pharmaceutical interventions (NPI) such as face masks and intensified hand hygiene may be effective in preventing influenza infection mainly in clinical settings or households, vaccination is considered the most effective way to prevent influenza [4]. If vaccination is not available, e.g., upon emergence of a new virus or in vulnerable groups of patients where vaccination is less effective, antiviral prophylaxis and treatment represent an additional option [5]. Most international and national public health institutions as well as medical and scientific societies recommend antiviral treatment of influenza at least for patients who are at high risk of developing serious influenza complications [6]. In contrast, anti-influenza drugs are used to treat large numbers of uncomplicated influenza cases in Japan [7]. Anti-influenza drugs are therefore an important part of a rational approach to epidemic influenza control, and are critical in planning a pandemic response [8].

This review summarizes the recommendations available in Germany for the use of antiviral drugs for prophylaxis and treatment of influenza considering specific circumstances and groups of patients in statements and guidelines of medical societies as well as the German pandemic preparedness plan.

## 2 Antiviral medicines

There are currently three classes of antiviral medicines authorized by the *European Medicines Agency* (EMA) for treatment and prophylaxis of influenza: the neuraminidase inhibitors (NAI), the M2 ion channel inhibitors (adamantanes), and the Cap-dependent endonuclease inhibitor baloxavir marboxil. The RNA polymerase inhibitor favipiravir is licensed in Japan for pandemic preparedness only (Table 1 (Tab. 1)).

Although combination therapy with two or more antiviral compounds might reduce the development of antiviral resistance and could increase antiviral potency especially in immunocompromised or seriously ill patients, all existing recommendations for influenza treatment relate to therapy with one antiviral drug (reviewed in [9]). In preclinical studies, the benefits of combination therapy over monotherapy were demonstrated [10], [11]. Controlled clinical therapy trials are difficult to carry out, not only for organizational reasons, but also because of the limited number of active antivirals. However, the planning of such studies is ongoing.

### 2.1 M2 ion channel blocker

The efficacy of adamantanes (amantadine and rimantadine) is limited by their inapplicability to influenza B viruses, as well as emergence and transmission of drug-resistant virus variants. Resistance to adamantanes emerges spontaneously due to natural polymorphisms or reassortment of influenza A viruses.

#### 2.1.1 Characteristics and mode of action

The M2 ion channel inhibitor amantadine blocks the release of influenza virus RNA from the endosome into the cellular cytoplasm. This effect is achieved with therapeutic dosage of the drug for influenza A viruses only, because the structure of the ion channel is different for influenza B viruses [12].

#### 2.1.2 Resistance

Since winter 2004–2005, adamantane-resistant influenza A(H3N2) viruses have spread worldwide. The emergence of the pandemic influenza A(H1N1) virus in 2009, carrying natural polymorphisms conferring reduced susceptibility to the adamantane class of antivirals (mainly the substitution S31N in the M2 ion channel), caused that currently all circulating human influenza A viruses are resistant to adamantanes. These findings rendered this class of drugs mostly ineffective [13]. In summer 2017, some adamantane-sensitive influenza A(H3N2) viruses encoding serine at residue 31 of the M2 protein were detected in Australia, suggesting the reversion back to the drug-sensitive phenotype more than 10 years after the resistant strain had emerged [14]. In Germany, approximately 25% (N=276) of the viruses detected by the German National Influenza Centre (NIC) during the 2018–2019 season were analyzed for M2 ion channel substitutions associated with resistance to the adamantane class of drugs. In all samples, the substitution M2-S31N and in addition in three viruses the molecular markers for resistance M2-L26F or M2-V27A/I were detected, suggesting that the circulating viruses of subtypes A(H1N1)pdm09 and A(H3N2) in Germany remain resistant to adamantanes [13].

#### 2.1.3 Recommendations

Due to the current resistance of circulating influenza A viruses, the clinical use of adamantanes is not recommended presently, but might be an option in case of pandemic spread of a new influenza virus sensitive to adamantanes.

### 2.2 Neuraminidase inhibitors

#### 2.2.1 Characteristics and mode of action

Neuraminidase inhibitors (NAI) are a class of antiviral agents acting against influenza A and B viruses by selectively inhibiting the viral neuraminidase and, thus, preventing the release of new viruses from infected cells as well as virus spread. Additionally, they affect viral invasion of the upper airways, and therefore suppress infection before virus entry into cells by blocking the cleavage of sialic acid moieties on the mucin by the viral neuraminidase [15]. Based on several clinical studies, NAI have been considered effective for prevention and treatment of influenza infections. In this respect, treatment shortens the duration of symptoms of influenza by approximately 0.5 to 1.5 days when started within the first two days after the onset of illness [6]. In critically ill, hospitalized patients, influenza treatment with NAI can be considered even when a longer time period has elapsed [16]. In 2014, NAI have been subject for critical discussions concerning their effectiveness and safety, as well as the appropriateness of stockpiling these drugs for use in future influenza pandemics. The World Health Organization (WHO) has moved oseltamivir from the core to the complementary list. However, the treatment of influenza with NAI is recommended for patients at higher risk for developing severe illness or infected with avian influenza viruses like A(H5N1) by WHO, CDC, and various national associations. An expert opinion from the European Centre for Disease Prevention and Control (ECDC) and national authorities, as well as national virological, medical, and therapeutical societies (Society for Virology (GfV e.V.), German Association for the Control of Virus Diseases (DVV e.V.), Paul Ehrlich Society for Chemotherapy (PEG e.V.)) confirmed earlier assessments that there is no significant new evidence from randomized control trials to support any changes to the approved indication and recommended use of neuraminidase inhibitors in EU/EEA member states (Table 2 (Tab. 2)). This position was consistent with guidance from WHO and other national public health organizations in the United States and Australia [17], [18], [19], [20].

#### 2.2.2 Neuraminidase inhibitors for influenza prophylaxis

**Oseltamivir** (Tamiflu™) and **zanamivir** (Relenza™) were approved and authorized by the *European Medicines Agency* (EMA) for prophylaxis of influenza in the European Union/European Economic Area (EU/EEA) in 2002 and 1999, respectively [21], [22].

Nosocomial influenza is a major threat since it can affect patients hospitalized for other, often severe underlying diseases. Consequently, the mortality is high. Nosocomial influenza is not a rare incidence and accounts for approximately 10–20% (range 5–45%) of all influenza cases in hospitals [23], [24], [25], [26], [27], [28], [29], [30].

Nosocomial outbreaks are frequently found and difficult to control. The virus might be introduced by asymptomatic/paucisymptomatic infected patients, visitors, or health care workers. Symptomatic persons are also a major source of infection in medical facilities with poor infection prevention awareness or in case of isolation measures breach. When controlling an influenza outbreak, it is challenging that the viral load is highest before symptoms are developed. Thus, the key measures usually taken in outbreak management such as isolation of symptomatic individuals are often not sufficient to stop transmission. In an outbreak situation, the use of neuraminidase inhibitors (NAI) might act as post-exposure prophylaxis in already infected individuals during the incubation period, or even as pre-exposure prophylaxis. NAI inhibit the viral replication and progression to disease sufficiently, especially when applied as early as for prophylaxis [31], [32], [33]. Therefore, the virus cannot be transmitted any longer [34]. In quite a number of studies, NAI could be shown to inhibit transmission effectively, and often to be the only measure that stopped an outbreak [35], [36], [37]. It must be kept in mind that the use of antivirals should be offered to all individuals involved in the outbreak, and not only to patients or to health care workers.

Patients with native, acquired, or treatment-induced immunodeficiency are at a particular risk for complications due to influenza. This might especially come true for the growing number of patients treated with biologicals such as rituximab [38]. It might be an important option in these patients to consider pre-exposure prophylaxis during the weeks with the highest infection risks to protect these highly vulnerable patients [39].

Usually, the dosage of NAI used for prophylaxis is only half of the dosage used for treatment. However, depending on the susceptibility of the virus strain, the prophylactic dose might even be doubled, e.g. for more resistant isolates that were sometimes found in avian influenza viruses [40]. In post-exposure prophylaxis, the therapy should cover 10 days. In pre-exposure prophylaxis, zanamivir is approved for 28 days, whereas oseltamivir is approved for up to 6 weeks in the general population, and for 12 weeks in immunosuppressed individuals. Sometimes, a double dose of NAI is discussed as off-label use in NAI-resistant strains. However, it should be kept in mind that resistant strains are rare at present; and even in case of resistance, the strains are often not resistant to all NAI. E.g., the H274Y A(H1N1) circulating in 2007–2008 was resistant against oseltamivir, but remained susceptible to zanamivir. Thus, the use of a double dose should only be taken into consideration in case no other options are availabe.

#### 2.2.3 Neuraminidase inhibitors for influenza treatment

Oseltamivir (Tamiflu™) and zanamivir (Relenza™) were also approved and authorized by EMA for treatment of influenza in the European Union/European Economic Area (EU/EEA) in 2002 and 1999, respectively [22], [41]. Additionally, EMA approved **peramivir** (Alpivab™) in April 2018 and intravenous **zanamivir** (Dectova™) in April 2019 for treatment of influenza [42], [43].

**Oseltamivir** is licensed by EMA for the prophylaxis and treatment of adults and children including full-term newborns. It represents a prodrug, and is available as oseltamivir phosphate in the form of capsules and as a powder for oral suspension. The compound is biotransformed by esterases in the intestinal tract and in the liver into the active metabolite oseltamivir carboxylate [22].

**Zanamivir** showed unsatisfactory oral bioavailability and was therefore mixed with lactose and inhaled as a dry powder. Zanamivir is approved by EMA for the treatment and prevention of influenza infections in patients starting from five years of age. In 2019, EMA approved intravenous (i.v.) zanamivir for the treatment of complicated and potentially life-threatening influenza caused by either influenza A or B viruses in adults and children from 6 months of age [43].

**Peramivir** (Alpivab™) was developed as an intramuscularly (i.m.) or intravenously (i.v.) applicable drug due to its poor oral bioavailability. In 2018, EMA approved peramivir as a single intravenous dose within 48 hours of the onset of influenza symptoms for the treatment of uncomplicated influenza in adults and children older than 2 years and withdrew the marketing authorization at the request of the marketing-authorization holder in December 2020 [44], [45].

**Laninamivir octanoate** (Japan: Inavir^®^) is approved in Japan only for influenza treatment and prevention of adults and children. Laninamivir octanoate presents as an octanoyl ester prodrug and showed therapeutic efficacy after one single nasal inhalation (long-acting neuraminidase inhibitor, LANI) [46]. After inhalation, laninamivir octanoate is absorbed by the respiratory tract’s epithelial cells and rapidly hydrolyzed to the active drug laninamivir [47], [48].

#### 2.2.4 Resistance

In Europe, antiviral susceptibility of influenza viruses to neuraminidase inhibitors is monitored by the European Centre for Disease Prevention and Control (ECDC) and the WHO/Europe Influenza Surveillance (EuroFlu). Since the influenza season 2006–2007, NAI-resistance monitoring is based on the reports sent by national influenza centers (NIC) to “The ECDC Surveillance System” (TESSy). The German NIC collects and weekly reports antiviral susceptibility data according to WHO guidance for determination of baseline and reduced antiviral susceptibility among circulating viruses [13], [49]. Susceptibility of circulating influenza viruses to the NAI oseltamivir, zanamivir, and peramivir was analyzed for about 500 viruses collected for the NIC’s influenza sentinel (44% A(H1N1)pdm09 and 56% A(H3N2)) in Germany during the 2018–2019 season. All but one tested viruses showed full in-vitro sensitivity to NAI. The NAI-resistant virus was collected approximately three days after initiation of oseltamivir treatment. It showed reduced susceptibility to oseltamivir and peramivir due to the substitution NA-H275Y. Further substitutions known to confer antiviral resistance to NAI were not detected [2].

The analysis of antiviral resistance data reported from twelve EU/EAA countries to ECDC, as well as the global analysis of antiviral susceptibility of influenza viruses sent from NIC to WHO Influenza Collaboration Centers revealed a low prevalence of viruses with reduced susceptibility to NAI (0.2%–0.5%) during the last ten seasons [50], [51].

#### 2.2.5 Recommendations

WHO, CDC, ECDC, and several national professional societies, e.g. Public Health England (PHE), the German Society for Pediatric Infectious Diseases (DGPI), and others, recommended to consider NAI for treatment of patients with confirmed or putative influenza who are hospitalized with medical conditions associated with an increased risk of influenza-related complications, or suffering from severe, complicated, and progressive course of disease (Table 2 (Tab. 2)) [6], [18].

### 2.3 Polymerase inhibitors

The RNA-dependent RNA polymerase (RdRP) of influenza viruses comprises three subunits: PA endonuclease, PB1 polymerase, and cap-binding PB2 subunit. PB1 synthesizes all viral RNA (mRNA, cRNA and vRNA). The cap-binding domain of the PB2 subunit (PB2cap) in the viral polymerase binds the cap of a host pre-mRNA molecule. PA represents a cap-dependent endonuclease. All three represent a promising target for anti-influenza drugs, but only PA and PB1 inhibitors are approved for treatment and prevention of influenza.

#### 2.3.1 Cap-dependent endonuclease inhibitor (baloxavir marboxil)

##### 2.3.1.1 Characteristics and mode of action

**Baloxavir marboxil** (Xofluza™) was licensed in Japan and the US in 2018, and recently in Taiwan (August 2019) for treatment of acute uncomplicated influenza in patients of 12 years of age and older who have been symptomatic for less than 48 hours. In January 2021, Xofluza was authorized in the EU for treatment and post-exposure prophylaxis of uncomplicated influenza by the *European Medicines Agency* (EMA) [52].

Baloxavir marboxil is an oral prodrug administered as a single dose which is hydrolyzed by the enzyme arylacetamide deacetylase to the active form baloxavir acid (BXA) [53]. A characteristic structural feature of the drug is represented by two oxygen atoms arranged in certain positions. These are able to enter into bivalent metal ion complexes. BXA inhibits the cap-snatching activity of the viral polymerase’s PA subunit, which prevents viral mRNA synthesis and thus virus multiplication. BXA acts as a chelator and complexes with the two 2-valent metal ions in the active site of the endonuclease, which are essential as cofactors for the efficacy of this viral enzyme [54]. Baloxavir demonstrated broad spectrum coverage, and additionally could provide an option for patients with infections caused by NAI- or M2I-resistant influenza virus with its novel mechanism of action [54].

According to the prescribing information for baloxavir marboxil tablets for oral use, the co-administration with polyvalent cation-containing products may decrease plasma concentrations of baloxavir and thus reduce its efficacy. Therefore, co-administration of baloxavir with polyvalent cation-containing laxatives, antacids, or oral supplements (e.g. calcium, iron, magnesium, selenium, or zinc) should be avoided.

##### 2.3.1.2 Resistance

Within the clinical trial, Capstone-1 variants of cap-dependent endonuclease (CEN) of the viral polymerase’s PA subunit with I38T/M/F substitutions emerged in 2.2% of patients infected with A(H1N1)pdm09, and 10.9% of those with influenza subtype A(H3N2) infection (Table 3 (Tab. 3)), suggesting a low barrier of resistance [55], [56]. The PA-I38X substitutions conferred an increase of TCID50 of baloxavir in yield reduction or other phenotypic assays, e.g. focus-reduction assay or high-content imaging neutralization test [57]. Results of *in vitro* viral comparative fitness assays suggest a detrimental effect of PA-I38X substitutions regarding the replicative capacity of viruses in MDCK cells [58]. On the other hand, a human-to-human transmission within a family cluster of A(H3N2) bearing the PA-I38T substitution was reported recently [59], suggesting retained replication and transmission capability of treatment-selected viruses. Therefore and for public health issues, monitoring of seasonal influenza viruses for baloxavir susceptibility by using phenotypic assays and genetic analysis for detecting molecular markers of resistance is being included into the influenza surveillance (USA [60], Japan [61], [62], [63], Asia-Pacific [64], Germany [2]).

##### 2.3.1.3 Recommendations

A systematic literature review and meta-analysis suggests that baloxavir marboxil demonstrated better or at least similar efficacy compared to other antivirals with a comparable safety profile [65]. Based on the results of several ongoing and completed randomized controlled clinical trials, baloxavir marboxil was found to be comparable to oseltamivir regarding secondary end points, including time to alleviation of symptoms of influenza-like illness. Baloxavir marboxil reduced the length of viral shedding by about 2 days when compared with placebo. Additionally, comparison with placebo showed a significantly reduced risk of developing influenza by 86% when used as prophylaxis (Table 3 (Tab. 3)) [55], [66], [67].

#### 2.3.2 PB1 polymerase inhibitor

##### 2.3.2.1 Characteristics and mode of action

**Favipiravir** (formerly known as T-705) represents a pyrazinecarboxamide derivative. Favipiravir is ribophosphorylated by host cell enzymes to the active metabolite, T-705 ribofuranosyltriphosphate (T-705RTP) [68]. T-705RTP mimics guanine and adenine, and acts as alternative substrate of the viral RNA-dependent RNA polymerase PB1. Its incorporation into progeny viral RNA during replication generates a high percentage of nonviable influenza viruses via lethal mutagenesis [69].

Favipiravir selectively inhibits a broad spectrum of RNA-dependent RNA polymerases, and thus inhibits the viral gene replication of influenza viruses and nine other RNA virus families [69], [70]. However, the clinical and virological effects of favipiravir in clinical trials in patients with uncomplicated influenza were modest, and there is evidence of teratogenicity and embryo toxicity [71], [72]. Today, favipiravir is approved for influenza pandemic preparedness in Japan only [73].

##### 2.3.2.2 Resistance

The favipiravir resistance-conferring amino acid substitution PB1 K229R in A(H1N1)pdm09 viruses could be generated in cell culture, but was not detected in clinical trials so far [74], [75].

##### 2.3.2.3 Recommendations

Favipiravir is part of the Japanese antiviral stockpile, and available in Japan only in case of an emerging new influenza virus, and may be used if the pandemic virus is resistant to other antivirals [76]. Its use is limited due to modest effects on the time to alleviation of influenza symptoms, and concerns of teratogenicity suggest that a more widespread use of favipiravir is unlikely [76].

## 3 Conclusions/Summary

Antivirals used as pre- and post-exposure prophylaxis represent an essential tool to stop outbreaks in institutions such as hospitals or nursery homes.

NAI, especially oseltamivir, remain the drug of choice for influenza treatment and prevention today. The low frequency of influenza viruses with reduced susceptibility to NAI over a ten-year period supports the use of NAI to treat influenza [50]. However, antiviral treatment of influenza should be restricted to severe illness in critically ill hospitalized patients and should be considered for in- and outpatients who are at higher risk [77]. Baloxavir marboxil presents a new therapeutic option with a single-dose oral regimen. Although it is a valuable addition to the available antivirals for treatment and prevention of influenza, novel antivirals are still needed. Therapeutic use of two or more anti-influenza compounds could increase clinical effectiveness and might be a promising approach for control of influenza in the future.

## Notes

### Acknowledgments

This review was supported by the Scientific Advisory Board for Antiviral Therapy of the German Association for the Control of Virus Diseases (DVV) and the Society of Virology (GfV) as well as the Section for Antiviral Therapy of the Paul Ehrlich Society for Chemotherapy (PEG).

### Competing interests

SD, BS, JT, OA, HF and MS declare that they have no competing interests.

BG received fees for talks and/or advisory board: Roche, Seqirus, Sanofi, GSK.

## Figures and Tables

**Table 1:**
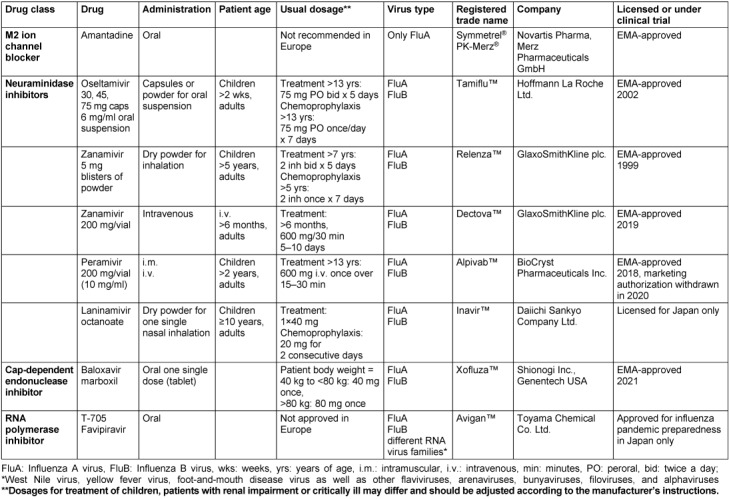
List of approved anti-influenza drugs (adopted and modified from [13], [78])

**Table 2:**
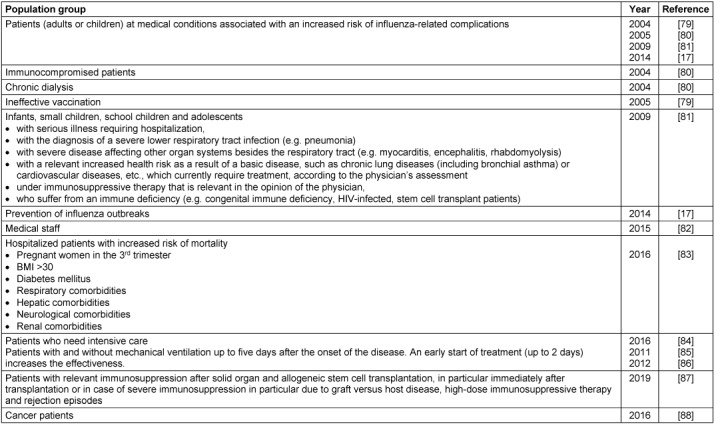
Treatment recommendations for treatment of suspected and/or laboratory-confirmed influenza available from German Guidelines and recommendations

**Table 3:**
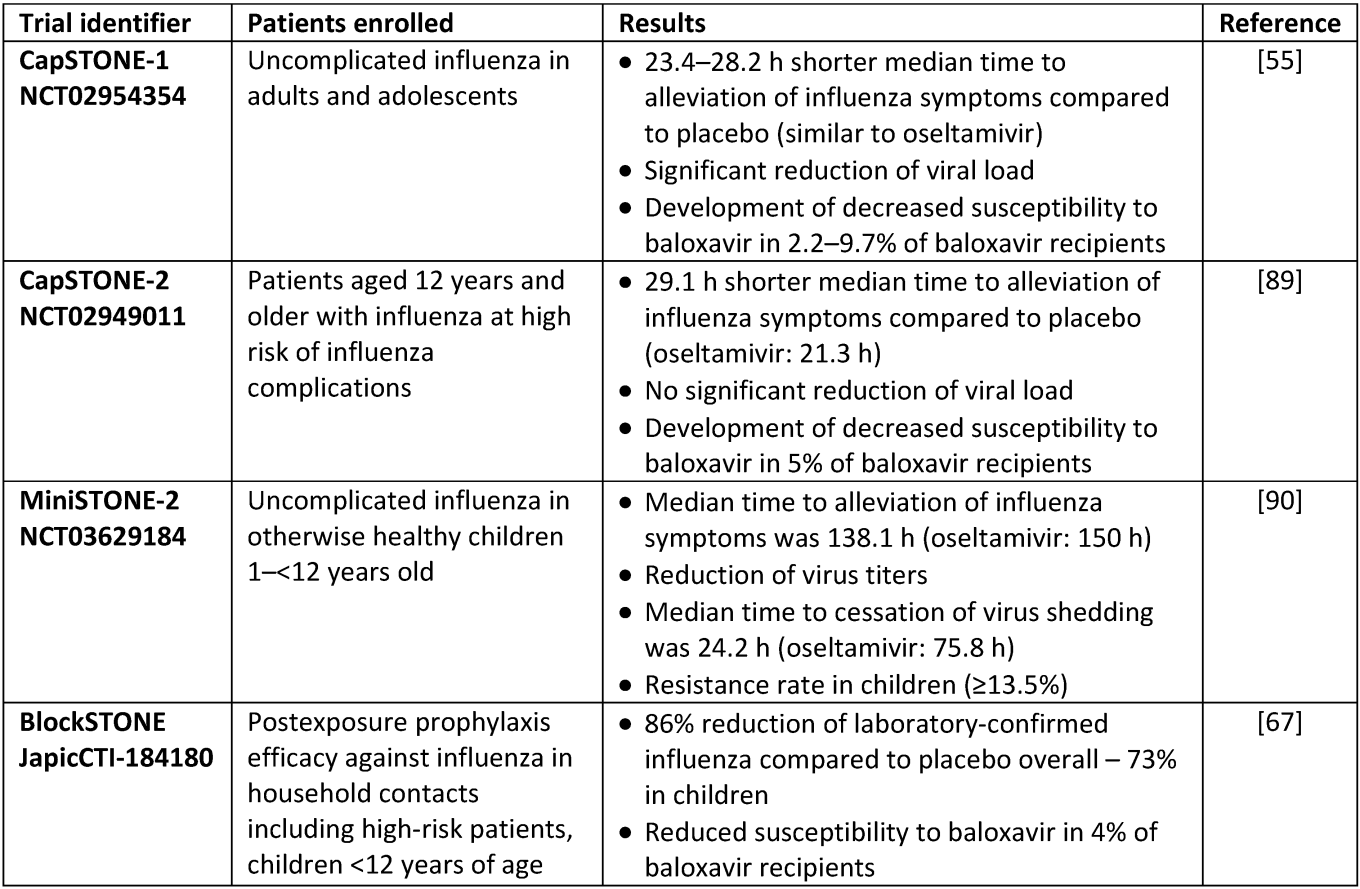
Summary of clinical trials phase 3 on influenza with baloxavir marboxil, oseltamivir and placebo
